# Harrison Noble, M.D., Heyworth, Ill.

**Published:** 1871-05

**Authors:** 


### Obituary.

Died.—At his residence near Heyworth, Illinois, on the 12th of August, 1870, Hon. Harrison Noble, M.D., in the 59th year of his age.

Dr. Noble graduated at the Ohio College of Medicine in 1847. Previous to this time he had been engaged in agricultural pursuits, and early in the history of McLean County was county surveyor. He was a man of great strength of mind and will, and a close student, and immediately upon his debut in medicine became prominent in the profession of the State. As President of the Illinois State Medical Society, he was one of its most honored and efficient presiding officers; he was regular in his attendance upon its meetings, and acted frequently and ably as chairman of important committees. At one time he made a special report upon typhoid fever, which was regarded as a very able effort. He was also a member of the American Medical Association, and frequently attended its sessions.

The doctor represented his district in the State Legislature two consecutive terms and was a prominent working member of that body. The then Governor of the State, declared in a public speech, that he owed more to him for valuable assistance than any other member of either house.

At the last gubernatorial canvass he was a prominent candidate for the nomination for Governor, by a number of the central counties of the State.

Personally, Dr. Noble was a man of splendid physique and commanding presence, a good advisor, and a true and faithful friend. By his death a void has been created in the circle of society he ornamented, and in the profession that he elevated by his counsels—but—

> "He gave his honors to the world again,
> His blessed part to Heaven, and slept in peace."

